# Promoting Quality of Life in Advanced Dementia Care: Reading Buddies Program as Service-learning

**DOI:** 10.21926/obm.geriatr.2102169

**Published:** 2021-04-30

**Authors:** Scott A. Trudeau, Megan E. Gately

**Affiliations:** 1. American Occupational Therapy Association; 2. Tufts University, Department of Occupational Therapy; 3. VA Bedford Health Care System, Geriatric Research Education and Clinical Center (GRECC)

**Keywords:** Occupational therapy, education, professional development, dementia

## Abstract

The Reading Buddies Program was developed as a service-learning component of an Occupational Therapy Practice with Older Adults course as a collaboration between Tufts University and the VA Bedford Health Care System. The purpose of this service-learning program was to challenge graduate students’ implicit biases and improve communication skills when working with older adults with significant cognitive impairments. Through this collaboration, occupational therapy students provided individualized, activity-based care to Veterans with advanced dementia. In this qualitative study, a total of 55 guided reflection papers submitted by students were analyzed using NVivo. Four major themes emerged: “I was a fish out of water,” “I finally took a risk,” “And then I thought, maybe I should give myself a little credit,” and, “I am still experimenting with how I feel,” illustrating student outcomes and perceived benefits of participation in the Reading Buddies Program. Each theme reflected the development of clinical reasoning which was the targeted impact. Outcomes confirm service-learning as an effective tool and suggest further use for academic programs, emphasizing the potential of creative partnerships to meet educational goals while providing valuable programming to vulnerable populations.

## Graphical Abstract

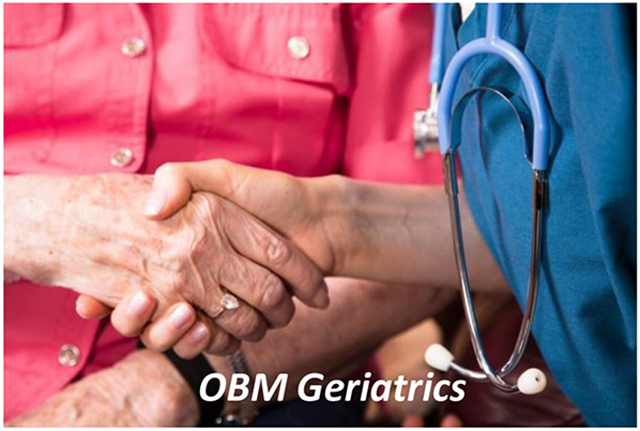

## 1. Serving Clients with Dementia

An estimated 5 million Americans have dementia, a number which is projected to expand to 15 million by 2050 [1]. Dementia poses multiple challenges to the health care team, and occupational therapy practitioners (OTs) are trained to assist clients and caregivers as they move through the stages of dementia and experience changes in functional status [2-5]. As the disease progresses, erosion of cognitive processes results in increasing dependence in activities of daily living (ADL’s) while orientation to person, place, and time becomes limited. Coupled with decreased motor and sensory functioning and loss of language, this complex array of symptoms requires specialty care, particularly in the latter stages, when the range of impairments often necessitate institutionalization [6]. Subsequently, there is a need for health care professionals who are prepared to meet the complex medical and psychosocial needs of this population. Yet there is a dearth of providers trained for the needs of older adults in general [7], a gap which is worse in dementia care [8].

Though the majority of those living with dementia live at home, late-stage dementia often necessitates long-term care. Long-term care settings typically require staff skills and abilities that differ from acute or outpatient settings. Strong communication skills are paramount, given the likelihood of frequent interaction with caregivers and necessary interprofessional team collaborations. A quality of life approach is necessitated by residents’ medical frailty, functional impairment, and cognitive decline [9]. Thus, in order to address quality of life needs, programs must ensure that adequate meaningful activities are provided[10]. Further, clinical training must adequately prepare health care professionals for this reality. However, many health care professionals, including occupational therapy practitioners, have minimal exposure to patients with progressive neurological conditions such as dementia [11] as part of their clinical affiliations.

## 2. Occupational Therapy Curriculum

Occupational therapy curriculum is structured to include didactic instruction and experiential practice to support students’ development of core clinical reasoning skills. Academic programs inherently tend to be classroom based and focus on more traditional, passive learning styles [12]. Students, therefore, exclusively depend on experiential learning opportunities to prepare them for practice. Required clinical preceptorships may not provide students with enough opportunity to fully develop entry-level competence in clinical reasoning [13], necessitating additional opportunities for skill development. Service-learning provides hands-on application of knowledge to bridge classroom learning with everyday practice in real-time.

## 3. Service-learning: A Prospective Solution

Service-learning is a form of experiential learning which offers students opportunities to apply course material while providing a form of community service to clients within actual clinical contexts [14-16]. These direct experiences afford unique educational opportunities that are difficult to replicate in the classroom, such as direct interaction with real clients, challenging interpersonal situations, and employee role modeling [16]. Exposure to these types of situations allows for development of knowledge and skills that are best honed in real-life practice contexts [13]. Service-learning allows students to explore their ability to form therapeutic relationships. Service-learning also reinforces didactic learning and increases students’ confidence and awareness of their own strengths and weaknesses as practitioners. Service-learning provides specific opportunities for students to explore their comfort and interest in working with certain populations. Simultaneously, all students are provided an opportunity for personal growth by challenging their implicit biases in a clinical context.

The approaching rise in the dementia population coupled with the intricacies of treating this complicated diagnosis demands attention from our academic training programs. Part of the solution may involve providing students with additional applied training while in school, particularly in real-world practice contexts [13]. Previous research has studied occupational therapy students’ experiences in service-learning with a wide variety of populations, including pediatrics [17], community-dwelling elders [15, 18], and individuals with mental illness [16]. Regardless of setting, students participating in service-learning demonstrated a growth in clinical reasoning. Specific improvements were found in skills related to interactive reasoning, including therapeutic use of self and the ability to form therapeutic relationships, and students reported that they had a better understanding of course material following their participation. Students were also noted to have increased self-confidence in their interactive reasoning skills, and a more realistic appreciation of their strengths and weaknesses as communicators. These findings reinforce the benefits of service-learning in occupational therapy academic programs. The growing need for a geriatrics workforce to meet the needs of clients with dementia further underscores the relevance of service-learning programs with this vulnerable population. Reading Buddies Program was created as a service-learning opportunity for students to gain hands-on experience while working with clients with dementia in a long-term care setting.

## 4. Reading Buddies Program

One of the challenges to providing activity programs in dementia special care units (DSCU) is the range of functional capacity across residents. Programs are typically directed to the more moderately functional, often not meeting the needs of the least and most functional. The recognition that a significant cohort (approximately 20%) of residents were typically excluded from group activities was of concern. This cohort came to be referred to as “VIPs” (Very Isolated Patients). The reason for their isolation was the difficulty in tailoring activities to their limited function within a group context. We speculated that individualized approaches would be more effective in one-to-one interactions but with limited staffing were not always able to provide this necessary support. Developing a structured intervention that could be deployed by students to this vulnerable population seemed advantageous for all.

This paper describes the Reading Buddies Program (RBP). RBP is an intergenerational service-learning opportunity at the VA Bedford Health Care System. RBP matched second year occupational therapy Masters-level students with Veteran residents of the dementia special care unit (DSCU). Students spent about 45 minutes each week reading aloud to residents for eight to twelve weeks of a fourteen-week semester. Providing cognitive stimulation and social interaction for residents with dementia, this program also provided hands-on clinical practice experience for occupational therapy students as the service-learning component of an older adult practice course. Outcomes reflect student perspectives of this service-learning experience.

Assessment and documentation are important aspects of student participation in the RBP, simulating actual practice demands. Students are matched with a resident to consistently read with throughout the program, barring unforeseen medical or other barriers. Each student attends RBP sessions with a partner who serves as observer during the session. With permission from the author, the researchers were able to adapt the Observational Measure of Engagement (OME) [19] to use as documentation during the reading sessions. Minor adaptations were made to ensure relevance to the program. The observing partner documented using the adapted OME during the reading session. The reading partner documented the session by writing a SOAP note. After a reading session, the two students switched roles and read to a second resident.

## 5. Methods

The purpose of this exploratory study is to evaluate student perceptions of their professional development achieved through service-learning. The research questions are:

- What themes were present in student reflections on RBP?
- What perceived benefits of service-learning did students identify?

After application to the Tufts University Institutional Review Board (IRB), this study was determined exempt from IRB review because the research utilized commonly accepted educational practices. The overall goal of this study is to examine assigned self-reflective work of graduate students who have taken part in the Reading Buddies Program. As part of the older adult occupational therapy practice course, students were assigned a cumulative reflection paper at the end of the semester. The assignment for the reflection paper was to garner student perceptions on how participation in RBP throughout the semester supported their development of an occupational therapy practitioner lens. In an effort to guide this reflection, students were asked to read Thomas Moore’s article, Body and Soul [20]. Questions^1^ were posed to prompt the students to consider the holistic impact of their experience during Reading Buddies Program. This summative assignment afforded students an opportunity to qualitatively assess their RBP experience, which had been monitored and recorded throughout the semester in various ways, as described above.

## 6. Analysis

Using a qualitative descriptive approach, as outlined by Hsieh and Shannon [21], detailed content analysis was performed using NVivo software. Fifty-five reflection papers from three semesters were analyzed for this study. Saturation was reached after 34 papers, but all papers were coded. Three reviewers met regularly in an iterative process to code the reflection papers, identify trends, perform code analysis, and finally cluster and collapse codes into four thematic areas. The researchers memoed throughout the process to record the evolution of the codes and kept an additional observation log to document throughout the project. To enhance the rigor of the methods, themes were compared to the course evaluation data, specifically, open text comments related to the service-learning component of the course. Evaluations were anonymous and submitted at the completion of the semester. By cross-verifying student perceptions of the RBP, the researchers were able to substantiate the findings in the study by triangulating the reflection data with course evaluation comments.

## 7. Results

After iterative analysis of the student reflection papers, four majors themes arose: “I was a fish out of water,” “I finally took a risk,” “And then I thought, maybe I should give myself a little credit,” and, “I am still experimenting with how I feel.” Highlighting the significance of the students’ experience in the Reading Buddies Program, themes describe an evolutionary process of gaining continual confidence in clinical reasoning skills. Each major theme emerged from codes that were organized into subthemes through the analysis process. The four themes are detailed below.

### 7.1 “I was a Fish out of Water”

The first major theme developed out of the codes was the novelty of working with a cognitively impaired geriatric population, along with students’ misconceptions, implicit biases, and tendency to favor work with pediatric populations. An initial trend the researchers noticed was for students to comment on their perceived strengths with a pediatric population. Of these students who wrote about their previously exclusive interest in pediatrics, many reflected that their participation in RBP made them reconsider what populations they were and were not willing to work with:

This semester has *been a time of growth for me* in terms of my occupational therapy lens, clinical reasoning and self-awareness…. I was a fish out of water. Put a child in front of me who is not yet verbal, is limited in function and cannot express his interests or preferences, and I feel competent and confident. However, interacting with an older adult with dementia illuminated new challenges and insecurities for me, and I was not sure how to approach this experience initially except with trepidation.

This is a powerful example of the importance of transferable skills to increase occupational therapy’s impact across populations and the lifespan. This student’s words also reveal an unease with dementia residents that she was able to problem-solve through during RBP, potentially increasing her comfort. As Renwick et al found, after having opportunities to work with older adults in a classroom setting, students reported more willingness and interest to work with older adults in other contexts [22]. This research supports the notion that providing hands-on opportunities to engage with older adults may help to meet workforce enhancement goals by challenging implicit biases and increasing competency with this growing population.

The majority of students started the RBP with some degree of uncertainty. The students were “nervous and hesitant,” they “felt unprepared to communicate with a nonverbal adult,” and were “anxious prior to starting.” Most students reported little experience with the population, with one student writing, “I’ve had virtually no experience working with Veterans or clients with dementia” and another noting, “I came into the Reading Buddies program with apprehension.” Many students expressed that participation in the RBP was their first time working with a nonverbal client, a client with dementia, a client who uses a wheelchair, or a client in a long-term care setting. The preponderance of students reflected that this was their first exposure to nonverbal clients and clients with dementia. Many students reported that during the semester, they recognized misconceptions they either believed to be true about the population or that were specific to their Veteran. This is consistent with Knecht-Sabres’ findings that “curricular renaissance” is needed in experiential learning opportunities in occupational therapy programs to ensure students have the preparation and confidence to transition into budding clinicians [15]. Learning opportunities such as these provide students with the chance to reflect upon their own belief systems within a context of self-directed learning.

### 7.2 “I Finally Took a Risk”

This theme developed from subthemes: book choice, therapeutic use of self, and clinical decision making. As students detailed their experiences, they discussed the evolution of a clinical decision-making process. In doing so, students revealed their understanding of the dynamic nature of occupational therapy practice and an increasing ability to be flexible during individual sessions:

I worried about gauging the level of difficulty in my efforts to find the “just right challenge” for [my Veteran], and it wasn’t until the second to last session that I finally took a risk and chose a book entitled *Joyful Noise: Poems for Two Voices*. I believe this experience laid the foundation for our final Reading Buddies session. During this session, I re-introduced the book that we previously had read entitled *Poems For Us All* by Lola Cooper. I turned to the section of poems that was about “Family” and [my Veteran] and I began to read the first two lines in unison. He continued to read aloud on his own for the remaining 42 minutes of the session. Every 1-2 minutes or so he would stop reading, however after I pointed to the line or stanza that he was on he would continue.

As poignantly outlined above, this student’s exploration of a novel genre, poetry, led to a meaningful and clinically significant session with her veteran whose behavior belied the oft-held notion that individuals with dementia are unable to sustain engagement in meaningful activities [23]. It is unclear if other factors contributed to the veteran’s positive response that day. However, the student’s ability to re-introduce a stimulus that had previously demonstrated moderate success and to successfully cue the Veteran to sustain engagement in reading, heightened his opportunity for independent activity while bolstering her confidence in her burgeoning clinical reasoning skills.

In another reflection, a student revealed the importance of attending to biological needs in order to facilitate performance, and an active grappling with the notion of engagement itself:

[My Veteran] seemed to respond best to rhythmic poetry readings with large and vibrant illustrations, so I spent several sessions reading Dr. Seuss books to her. I soon realized that some of [my Veteran’s] fidgeting stemmed from needing to have her undergarments changed, and I learned how to time our sessions around the bathroom rotation schedule that the nurses kept. I also learned that [my Veteran] was not necessarily asleep when her eyes were closed, as reminded by [my Veteran’s] occasional smile and chuckle that often preceded her opening her eyes.

As an example of a student moving away from solely procedural reasoning, this student begins to consider the complexities of her Veteran’s unspoken needs and how they could be affecting participation in the activity. Mitchell and Unsworth identified a key difference between expert and novice occupational therapists as the ability of experts to fluidly employ different types of reasoning throughout one session. Novices consistently relied on procedural reasoning and rigidly stuck to that thinking [24]. Students in the Reading Buddies Program had the opportunity to be creative and problem solve in a supported learning environment, which allowed them to demonstrate emerging skills as practitioners.

Risk-taking was reflected not only in directed interactions with Veterans but also exploring the role of occupational therapy on interprofessional teams. One student reflected,

It was helpful for me to communicate with the social worker about [my Veteran] to gain a better sense of his occupational profile (including his strengths, limitations, interests, and values). She explained that [my Veteran] used to have an interest in gardening but I learned that his hobby did not translate to an enjoyment in reading about gardening.

Within the context of the clinical environment, the RBP afforded students multiple opportunities (risks) to test their capacity to function clinically. In spite of the many opportunities for risk-taking, students who were most willing to be vulnerable benefited most.

### 7.3 “And then I Thought, maybe I should Give Myself a Little Credit”

Student reflections also revealed burgeoning self-efficacy, which has been found to relate to students’ perception of the meaningfulness of their clinical educational experience [25].

Sometimes I would catch myself thinking that maybe I was missing out on a particularly “challenging experience” that I heard so many classmates discuss. Other times I would think that I lucked out being assigned to [my Veteran], because she was, for the most part, pleasant throughout all of our interactions and I grew attached to our relationship. And then I thought that maybe I should give myself a little credit for my experience. I didn’t just go in blindly each week and hope for the best with [my Veteran]. I thought about what worked and what didn’t work in our sessions and I tried to build on that week by week. I learned to anticipate certain behaviors that I could expect or certain comments that she would likely make. Through that, I believe I established a genuine rapport with her, even though I wouldn’t expect her to remember me from week to week.

Providing students with the supported opportunity to take risks and grow as clinicians led to universally talked about improvement in confidence and/or perceived efficacy. Experiential learning in occupational therapy allows students to confirm their choice to pursue a career in the field as they apply their newfound knowledge and allows them to deepen their understanding of the role of the occupational therapist [17]. RBP offered a chance for students to integrate learning from their various academic courses.

Throughout my time in school, I have often been told how to respond to tough questions, but there is no way to anticipate the uncomfortableness that a tough question might bring up until actually being in that situation. For me, whenever [my Veteran] talked about wanting to go home or would perseverate on needing to find someone, I, in theory, sort of knew how I should respond, but in actuality, a response to such an emotionally provoking question or statement was not easy. It didn’t become easier over the course of the RBP, but at least I know what feeling uncomfortable in that context is like, and now I can think more critically about responding to tough questions with sincerity in the heat of a clinical encounter.

There are some clinical situations that are impossible to simulate in the classroom. Students in RBP experienced this as they learned to navigate in their sessions with their Veterans with advanced dementia.

### 7.4 “I am Still Experimenting with How I Feel”

This theme emerged from students’ reflections about their performance during RBP sessions, including emotional reactions. Students wrote of moments when they “felt silly” or were “hyper aware,’” describing that sometimes they were “frustrated” with themselves or their Veterans and found that rapport building can be a “slow process.” They wrote about sessions that left them with more questions than answers, while describing their development of clinical reasoning skills:

I learned to appreciate the significance of small gestures, sustained eye contact, and flickers of the hand. In attempts to engage her, we gradually become more frustrated at the predicament of interacting with a resident who made erratic eye contact and offered unintelligible comments to us. We remain curious about her personality and history, and accept that we will never really know the “true” [Veteran].

Opportunities to explore “reflection-in-action,” a process which involves a focused examination of particular situations and attempt in the moment to address it, is central to the clinical reasoning process of occupational therapy practice [26], and a valuable component of the RBP service-learning experience. The students did not leave the Reading Buddies Program as experts or feeling like experts. Rather, they made progress in their journey towards becoming clinical experts. Experts demonstrate confidence and are able to use conditional and interactive reasoning in sessions [24]. The growth from a novice to an expert requires ample experiential knowledge and for the students in RBP, this was one of their first steps to achieve that.

Students were also afforded an opportunity to test their own perceptions and comfort with certain aspects of client care, such as the often-intimate nature of practice. Reinforcing the benefit of exposure to client experiences, one student’s reflection eloquently described an exploration of therapeutic use of touch-an oft-cited but hard to operationalize construct within clinical practice:

I am still experimenting with how I feel about ‘romantic’ or ‘intimate’ touch with clients. It makes me uncomfortable when I see it happen, but I understand it can also be positive or negative. I don’t want to rebuke a client and I don’t want to shame them if they do show affection. It was nice to have some positive modeling by my peers and staff at the VA. I can be playful with clients and still limit-set what I feel comfortable with.

Sexuality and intimacy are often problematized amongst long-term care residents, particularly those with cognitive impairment [27, 28]. Therefore, students’ ability to critically examine their own notions of this aspect of client care by practicing-in-context demonstrates the value of this program in supporting students’ development of their clinical boundaries.

## 8. Discussion

The Reading Buddies Program targeted institutionalized Veterans with progressive dementia, a challenging population for the most seasoned professionals. Focusing on engagement with the most difficult to engage residents (VIPs) offered students rich opportunities to challenge and advance their professional identities. Providing a nuanced and detailed look at students’ experiences within a hands-on service-learning opportunity, these qualitative findings from the Reading Buddies Program offer insight into the development of students’ clinical reasoning skills and how RBP provided an opportunity to challenge implicit biases. Findings also reveal students’ increasing comfort with a population with unique and complex care needs and their developing recognition that engagement and promotion of quality of life are possible within this clinical context. Structured provision of meaningful activity is critical in this population even if difficult to operationalize [29]. Service-learning opportunities like this may allow for structured one-to-one engagement that is not feasible with existing staffing.

As an exemplar of a program designed to provide students with opportunities to problem-solve challenging situations in the moment and to participate in self-directed learning, RBP is also an educational model that may prove to be of particular relevance as we strive to meet the workforce capacity needs of a growing aging population [30]. Research supports the use of service-learning in academic programs [12]. It stands to reason more academic programs will integrate this as an educational tool in the near future, necessitating a conversation about how there is a need to think critically about what components to include when building these opportunities. Reading Buddies Program paired students with one Veteran over the course of the semester, allowing them time to develop important skills for after graduation as they: built rapport, gained exposure to long term care settings, improved observation and documentation skills, and, practiced preparing for a session only to change it in the moment. The professional development of students in RBP is noted across themes in this analysis. Students were not gambling or blindly guessing, but instead this program allowed them to apply their clinical instruction and begin to build their clinical skills which will serve them in their years to come as evolving practitioners.

## 9. Conclusions

As the population with dementia continues to expand, so does the need to train health care providers to address the complex and ever-changing needs of clients with dementia and their caregivers. However, evidence shows a lack in health care providers trained to work with clients with dementia and their caregivers [31]. While the presence of OT practitioners in long-term care and skilled nursing facility settings has grown [32], many OT practitioners report a lack of knowledge about interventions for dementia [33, 34], highlighting the need for more training and education in this area. Further, according to findings by these authors, only a small portion of OTs report working with clients with advanced dementia, a population with distinct needs, given the symptomology and increased dependency and inability to initiate meaningful activities [9, 35]. Experienced practitioners working with clients in the advanced stage also report a gap between interventions and their perceived effectiveness, highlighting the need for more training in this area [9].

Regarding training of health care providers in dementia care, little evidence supports programming for occupational therapy students to experience working with clients with dementia. One study of an interprofessional education (IPE) program afforded occupational therapy graduate students the chance to work with students from physical therapy and speech-language pathology, reporting student satisfaction with knowledge gained [36]. In a study of structured educational programs that include exposure to clients with dementia, all but one program was for medical students [11]. Opportunities like the Reading Buddies Program offer students an opportunity to develop and explore clinical reasoning skills with a population that requires a holistic, interdisciplinary approach to support quality of life. The Reading Buddies Program is an exemplar of the clinical and educational benefits of such a learning opportunity.

## Footnotes

1 Reflect on your service-learning experience. As discussed in class this portion of the assignment is specific to your Reading Buddies experience. It should be a general description and reflection of your experience(s).

- What do you consider your strengths as an occupational therapist? …weaknesses?
- In the past few years, how has your personal understanding of occupational therapy changed?
- How do you see yourself embodying the art and science of the profession?
- What vulnerabilities do you bring to your work? How will these enhance or block your therapeutic stance?
- How will you ensure that you maintain an appreciation of **WHOLE** people with whom you work? Do you anticipate that this will change over time?
